# New optical coherence tomography biomarker for diagnosis of acute optic neuritis in multiple sclerosis

**DOI:** 10.1016/j.heliyon.2025.e42114

**Published:** 2025-01-22

**Authors:** Hend Mohammed Safwat, Sanaa Ahmed Mohamed, Ahmed Hassan Elsheshiny

**Affiliations:** a Ophthalmology Department, Faculty of Medicine for Girls, Al-Azhar University (Cairo), Al-Zhraa University Hospital, Ophthalmology Building, Abasyia, Cairo, Egypt; b Ophthalmology Department, Faculty of Medicine for Girls, Al-Azhar University (Damietta), Egypt; c Neurology Department, Faculty of Medicine, Al-Azhar University (Cairo), Egypt

## Abstract

**Objectives:**

Is imaging of retrolaminar optic nerve, during attack of acute optic neuritis in multiple sclerosis valuable?

**Methods:**

This is a prospective observational Case series study. Twenty two patients recruited from Al-Azhar University Hospitals and Charity Eye Centre (El-Mustafa Eye Centre, Cairo); from October 2022 to February 2024. The patients were referred; as, they had developed episode of acute optic neuritis. Full ophthalmic and neurological examinations were done for all patients within 2 weeks of acute optic neuritis. Imaging of peripapillary retinal nerve fiber layer by conventional spectral domain optical coherence tomography (OCT), and retrolaminar part of optic nerve by enhanced depth imaging OCT were done for both acute optic neuritis eyes and the fellow eyes.

**Results:**

A total of 44 eyes of 22 MS patients (18 females, 4 males) with the mean age of 30.54 ± 9.65 years were included in this study. The best corrected visual acuity (BCVA) was significantly less in the optic neuritis eyes (median = 0.30) than in the fellow eyes (median = 0.70), *p* = 0.007. Homogeneity of retrolaminar optic nerve tissue was altered in the optic neuritis eyes. OCT showed myelin aggregates as round or oval hyper-reflective foci in the optic neuritis eyes (95 % confidence interval: 2.90 [2.19–3.61]), that were not presented in the fellow eyes, *p* = 0.000.

**Conclusion:**

Using enhanced depth imaging OCT during acute attack of optic neuritis revealed retrolaminar hyper reflective foci as a new biomarker.

## 1 Introduction

Multiple sclerosis (MS) is the most frequent inflammatory demyelinating disease of the Central Nervous System, and a leading cause for permanent neurological disability in young adults. Relapsing remitting MS (RRMS): the most common form of MS is characterized by intermittent attacks of symptoms (relapses); followed by a short or long period of no clinical attacks (remissions) [1,2].

Multiple Sclerosis International Federation (MSIF) estimated number of people with MS worldwide has increased to 2.8 million in 2020. The 2020 global prevalence was 35.9 per 100,000 people [3].

Optic neuritis (ON) is the most common optic neuropathy in young adults; with an incidence rate of 3–5 per 100,000 persons every year. ON is common in the course of MS. Up to 70 % of patients with MS having an acute ON during their course [4,5].

Typical acute ON is clinically diagnosed by a loss of vision that develops over days, dyschromatopsia, visual field loss, and pain that is often exacerbated by eye movements. MRI fat saturation techniques help to visualize gadolinium enhancement of the optic nerve during inflammation [6].

Because ON requires different acute and long-term treatment strategies, earlier diagnostic stratification is essential for the treatment decision; thereby, improves visual outcome.

A Study in USA included of the 122 patients who were referred for ON; founded about 40 % true and 60 % false diagnosis. The errors were in history taking especially in pain item, overweighting of signs and inability to put differential diagnosis [7].

Petzold et al. who is a panel of 101 international experts consisting of neurologists, ophthalmologists, neuro-ophthalmologists and neuro-radiologists developed new diagnostic criteria for acute ON. The definite diagnosis is implied if conventional clinical criteria with one paraclinical test are fulfilled. Those paraclinical tests are MRI, OCT or serological test [8].

The use of MRI in diagnosis of acute ON is established with bias in its sensitivity of diagnosis. The sensitivity ranged from 20% to 80 %. The focal enhancement of optic nerve during acute attack in MS-related ON is not apparent in each case. MRI enhancement is better elicited in other types of ON as in neuromyelitis optica spectrum disease (NMOSD) or anti MOG spectrum, because of longitudinal or bilateral affection. Also, the sensitivity of MRI for the intraorbital region of optic nerve is poor [8,9].

Optic nerve structural changes in the course of ON have been studied over the last 2 decades by advances in optical coherence tomography (OCT). However, OCT peripapillary retinal nerve fiber layer (pRNFL) thickness in acute stage may be normal or swollen; that lowers its value in this stage. Hence, the need for pararclinical test in the definite diagnosis of acute ON; that reduces misdiagnosis and titrate pharmacological intervention.

Objective, rapid, non invasive and safe diagnostic test for acute ON is a challenge for researchers. We introduce a new method by using OCT during acute ON in MS patients. Our novel is imaging of retrolaminar optic nerve with higher resolution enhanced depth imaging OCT (EDI-OCT). This is based on optical prosperities of myelin debris in acute inflammation.

### 1.1 Myelin optical properties

The myelin is composed of densely packed lipids and proteins; with a high refractive index; resulting in strong scattering in aqueous media. In addition, the myelin sheath exhibits strong optical birefringence which is a consequence of the anisotropic structure of the lipids [10].

Because axial and lateral resolutions of SD-OCT (5–30 μm) are more than an individual axon and its myelin sheath thickness (0.3–5 μm), this is not sufficient to capture details of optic nerve. Optic nerve axons and their myelin sheaths appear as homogenous iso-reflective tissue. In acute inflammation: demyelination process occurs, and myelin debris is formed. Homogeneity of optic nerve is altered, and OCT can show myelin aggregates as round or oval hyper-reflective foci due to a differential reflectivity.

## 2 Patients and methods

This work is a prospective observational Case series study. Twenty two patients recruited from Al-Azhar University Hospitals and Charity Eye Centre (El-Mustafa Eye Centre, Cairo), from October 2022 to February 2024. The study was approved by the Ethics Committee of the Faculty of Medicine for Girls, Al-Azhar University. We also adhered to the Tenets of the Declaration of Helsinki. Each participant was informed about this study, and his or her approval to participate was documented.

All patients were diagnosed as MS: either clinically isolated syndrome (CIS), or RRMS. The diagnosis of MS was made in the neurology department; based on McDonald criteria. Additional diagnostic tests were performed as needed; including the examination of cerebrospinal fluid, aquaporin 4 (AQP4) and myelin oligodendrocyte glycoprotein (MOG) antibodies. The patients were referred to our neuro-ophthalmic clinic, when they developed episode of acute ON. All patients were examined by 2 neuro-ophthalmologists as regard the best corrected visual acuity (BCVA) with Snellen decimal chart, color vision test using Ishihara test, red desaturation test using mobile app (Smart Optometry – Eye tests, version 4.1.4), pupil examination by swinging flash test, ocular motility, saccades in horizontal and vertical gazes, anterior segment by slit lamp, and fundus by slit lamp biomicroscopy using 90 D lens. Acute ON diagnosis was made clinically with those features: monocular diminution of vision within days, preceded by retrobulbar and ocular movement pain, low contrast sensitivity, color desaturation, relative afferent papillary defect (RAPD), and fundus examination that consistent with ON.

The decision about introducing intravenous steroid and/or changing MS modifying drugs; is requiring a diagnostic tool for documentation of acute attack. The only one available is contrast T1 magnetic resonance imaging (MRI) that reveal focal enhancement of retrobulbar optic nerve. However, MRI finding is not consisting in acute ON of RRMS, and likely to be over diagnosed.

### 2.1 Imaging

Conventional SD-OCT pRNFL and EDI-OCT imaging were done by 2 OCT devices for both eyes of all patients within 2 weeks from acute ON beginning. SD-OCT; Spectralis; Heidelberg Engineering Co., Heidelberg, Germany) was performed for both eyes of each participant. Serial vertical or horizontal B-scan images of the ONH were obtained using a method: The OCT device was set to image a 15° (vertically) × 15° (horizontally) rectangle centered on the optic disc. The enhanced depth imaging and high sensitivity modes of the Spectralis SD-OCT were on. This rectangle was scanned with 146 sections, with an interval between adjacent sections of approximately 30 μm. The good quality images were considered for analysis, and the poor images were rejected based on OSCAR IB criteria [(O) = obvious problems including violation of the protocol; (S) poor signal strength defined as <15 dB; (C) wrong centration of scan; (A) algorithm failure; (R) retinal pathology other than MS related; (I) illumination; and (B) beam placement]. To enhance lamina cribrosa (LC) and retrolaminar optic nerve visibility at the EDI-OCT scans; image contrast was increased to a higher level, and image colours were switched (black and white) as needed using the device software. We take frames with good resolution on temporal side retrolaminar; to avoid vascular shadowing nasally and LC density masking effect centrally. The LC was defined as the distance between the anterior and posterior borders of the highly reflective region at the vertical center of the ONH in the EDI OCT cross-sectional B-scans. Retrolaminar optic nerve tissue is homogenous and less reflective than LC.

The 2nd device used in this study was Angiovue/RTvue-XR Avanti RTVue-XR Fourier-domain OCT instrument (Optovue Inc., Fremont, CA, USA). Its EDI mode is only single line of imaging. So, we used manual mode of raster imaging to make zero delay line at chorio-retinal instead of vitreo-retinal interface. Hence, imaging of retrolaminal tissues with multiple B sans and high resolution could be done. The image quality was assessed. According to manufacturer's recommendations images with signal strength index less than 35 were excluded.

The OCT images were examined by 2 different examiners: 1st was optic neuritis status blinded and the other was non blinded. The OCT images were chosen when they had agreed: there are hyper-reflective foci and their numbers.

### 2.2 Statistical analysis

Statistical analysis was performed using SPSS version 20 (SPSS Inc., IBM, Chicago, IL, USA). The Shapiro-Wilk test for normality and the Levene tests for homogeneity of variance were performed on the data set. Descriptive nominal data was expressed as percentages. Descriptive numerical data was expressed as mean, standard deviation, minimum, and maximum.

Testing variance of BCVA, global pRNFLT, temporal pRNFLT, and retrolaminar hyper-reflective foci in the acute ON eyes group and the fellow eyes group were done. We used independent student t or Mann-Whitney test according to normality of data distribution. Data is expressed as mean (standard error = SE) or median (quartiles 25%–75 %), depending on the distribution. *P* < 0.05 was considered significant.

Testing correlation of our new variable (retro laminar demyelination foci numbers) to BCVA, global RNFLT, and temporal RNFLT; was done by using Pearson's coefficient test. Correlation was significant at the 0.05 level (p-value).

NB; 1 demyelination Plaque was transformed to 2 foci for easier statistical analysis.

## 3 Results

A total of 44 eyes from 22 MS patients (18 females, 4 males) with the mean age of 30.54 ± 9.65 years were included in this study. They were included in 2 groups: 22 eyes in the acute ON eyes group, and 22 in the fellow eyes group. The side of acute ON eyes was 13 right (OD: Ocular Dexter), and 9 left (OS: Ocular Sinister). The BCVA was 0.36 ± 0.20 in the acute ON eyes group, and 0.62 ± 0.30 in the fellow eyes group. The colour desaturation was elicited in all acute ON eyes group (100 %), whereas RAPD in 19 acute ON eyes (86.36 %). Optic nerve head appearance in the acute ON eyes was variable from normal appearance to cupping as shown in Table 1. The patients’ report of previous attack (s), either in acute ON eyes or the fellow eyes is shown in Table 1.

**Table 1:** Descriptive data of MS patients with acute optic neuritis.

Variables	N = 22
age	30.54 ± 9.65 (16–45)
gender	Females: 18/22 (81.81 %)
	Males: 4/22 (18.18 %)
BCVA	Acute ON eyes group: 0.36 ± 0.20 (0.05–0.08)
	Fellow eyes group: 0.62 ± 0.31 (0.05–1.00)
SE	Acute ON eyes group: 0.17 ± 0.97 (-2 – 2)
	Fellow eyes group: 0.10 ± 0.93 (-2 – 2)
The side of acute ON eye	OD: 13/22 (59.09 %)
	OS: 9/22 (40.90 %)
Presence of RAPD	Positive in 19/22 (86.36)
Colour desaturation	Positive in 22/22 (100 %)
ONH appearance of the acute ON side	Temporal pallor: 9/22 (40.90 %)
	Normal: 9/22 (40.90 %)
	Swollen: 3/22 (13.63 %)
	Cupping: 1/22 (4.54 %)
No of previous attacks	Acute ON eyes group: 0.59 + 0.66
	Fellow eyes group: 0.72 + 0.88

BCVA: best corrected visual acuity, SE: spherical equivalent, OD: Ocular Dexter, OS: Ocular Sinister, RAPD: relative afferent pupillary defect, ONH: optic nerve head, ON: optic neuritis.

Both BCVA and OCT global pRNFLT variances, in both acute ON eyes group and the fellow eyes group, showed non normal distribution (Table 2). The BCVA was significantly less in the acute ON eyes group (median = 0.30) than in the fellow eyes group (median = 0.70), *p* = 0.007. The OCT global pRNFLT was insignificantly higher in the acute ON eyes group (median = 97.50) than in the fellow eyes group (median = 96.50), *p* = 0.681.

**Table 2:** Variance of BCVA and global pRNFLT between the optic neuritis eye and the fellow eye.

Variables	Median (IQR)		*P*-value
	Acute ON eyes group (N = 22)	The fellow eyes group (N = 22	
**BCVA**	**0.30(0.30)**	**0.60(0.70)**	**0.007**
**Global pRNFLT**	**97.50(24.50)**	**96.50(27.75)**	**0.681**

IQR: interquartile range, ON: optic neuritis, pRNFLT: peripapillary retinal nerve fiber layer thickness.

An independent-samples *t*-test was done to determine if there was difference belong to the temporal pRNFLT in the acute ON eye and the fellow eye groups. The temporal pRNFLT was insignificantly different in the two groups as shown in Table 3 (*p* = 0.826).

**Table 3:** Variance of temporal pRNFLT between the optic neuritis eye and the fellow eye.

	Mean (SD)		Difference (95 % CI)	*P*-value
	Acute ON eyes group (N = 22)	The fellow eyes group (N = 22)		
**Temporal pRNFLT**	**60.45(16.90)**	**61.63 (18.57)**	**−1.18 (-11.98, 9.62)**	**0.826**

CI: confidence interval, SD: standard deviation.

### 3.1 New OCT biomarker

**Image 1:**
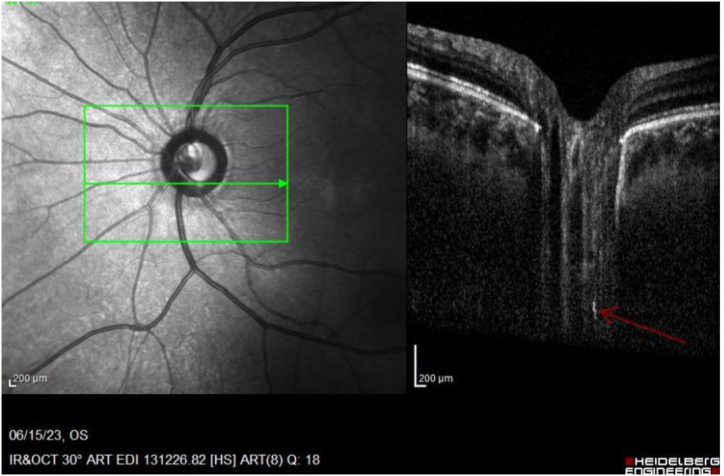
EDI-OCT of left eye ONH (horizontal raster) shows hyper-reflective plaque in retorlaminar tissue (red arrow).

In acute inflammation: demyelination process occurs and myelin debris is formed. Homogeneity of optic nerve is altered, and OCT can show myelin aggregates as round or oval hyper-reflective foci; some of them may beside each other forming elongated hyper-reflective plaque (Image 1, Image 3, Image 4)**(supplementary video).** Those retrolaminar hyper-reflective foci were only elicited in acute ON eyes groups, and not in the fellow eyes groups (Table 4) with significant results: (median + 95 % CI; 2.90 [2.19–3.61], *p* = 0.000) (Table 5).

**Image 2:**
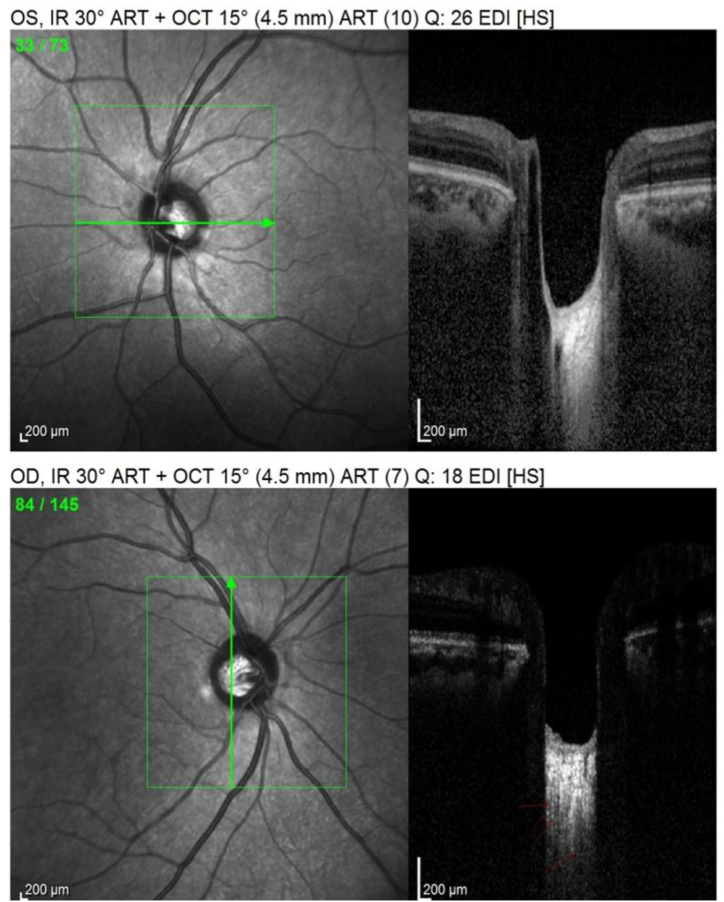
EDI-OCT of both ONH for one patient: upper one is the normal fellow eye that shows homogeneity of the retrolaminar tissue; the lower one of the acute optic neuritis eye that shows heterogeneity and hyper-reflective foci in the retrolaminar tissue.

**Image 3:**
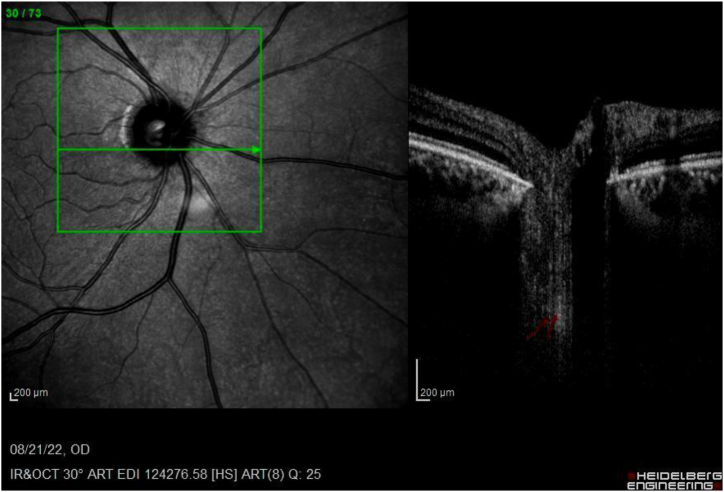
EDI-OCT of right eye ONH (horizontal raster) shows hyper-reflective plaque in retorlaminar tissue (red arrow).

**Image 4:**
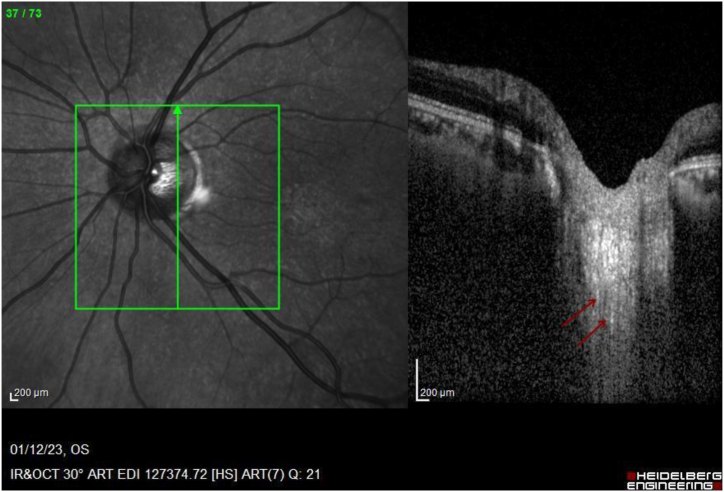
EDI-OCT of left eye ONH (horizontal raster) shows hyper-reflective foci in retorlaminar tissue (red arrow).

**Table 4:** Descriptive data of retrolaminar findings in 22 MS patients by EDI-OCT.

Patients	The acute ON eyes group	The fellow eyes group
1	3 demyelinating foci	no
2	2 demyelinating foci	no
3	3 demyelinating plaques	no
4	6 demyelinating foci	no
5	2 demyelinating plaques	no
6	5 demyelinating foci	no
7	1 demyelinating plaque	no
8	3 demyelinating foci	no
9	no	no
10	2 demyelinating foci	no
11	1 demyelinating plaque	no
12	2 demyelinating foci	no
13	1 demyelinating plaque	no
14	1 demyelinating foci	no
15	2 demyelinating plaque	no
16	5 demyelinating foci	no
17	3 demyelinating foci	no
18	1 demyelinating plaque	no
19	2 demyelinating plaques	no
20	1 demyelinating foci	no
21	1 demyelinating plaque	no
22	3 demyelinating foci	no

**Table 5:** frequency analysis of retrolaminar myelin debris foci in both groups.

	Mean (SD)		Difference (95 % CI)	*P*-value
	Acute ON eye (N = 22)	The fellow eye (N = 22)		
**Frequency of myelin debris**	**2.90 (1.60)**	**0.00**	**2.19**–**3.61**	**0.000**

CI: confidence interval, SD: standard deviation.

The new OCT biomarker (retrolaminar demeylinating foci) and BCVA showed inverse correlation with significance (p = 0.01). Correlations of retrolaminar foci and OCT pRNFLT variables (Table 6) were inversely related, with significance, in temporal pRNFL quadrant (*p* = 0.01), and non significance in global pRNFL thickness (*p* = 0.26).

**Table 6:** Correlation between number of retrolaminar myelin debris foci in acute ON eyes and some variables.

	No of retrolaminar myelin debris foci	
	r	*p*-value
**BCVA**	**−0.518**	**0.013**
**TEMPORL pRNFLT**	**−0.526**	**0.012**
**GLOBAL pRNFLT**	**−0.251**	**0.260**

r: correlation coefficient.

## 4 Discussion

As regard age of patients: we found its mean is 30.54 ± 9.65 years old. This result compared to the atlas of MS survey; is more or less the same. They found high variability in the age, but their mean globally at 2020 was 32 (20–44 years) [3].

Globally, females are twice as likely to have MS as males, and this is consistent with both prior editions of the Atlas. However, the ratio of women to men is as high as 4:1 in some countries; similar to our study result [3].

As regard BCVA: the loss was moderate as previous ONTT study. They found high-contrast visual acuity loss is moderate; with the majority of patients having acuity better than 20/200. Conversely, optic neuritis associated with NMOSD or MOG-IgG often presents with severe vision loss worse than 20/400 [11].

The presence of RAPD in the most of our cases strengthens the clinical diagnosis of acute ON. The absence of RAPD should always raise a diagnostic concern, unless the patient has bilateral involvement or a history of optic neuropathy in the other eye [11,12].

We found variable appearance of ONH in the fundus examination. Temporal pallor or cupping may be explained by previous attacks of ON in the same eye. In idiopathic optic neuritis: the funduscopic examination is typically normal with less than 25 % of patients presenting with disc edema [12].

Our results: the acute ON eyes global pRNFLT are thicker than the fellow eyes in acute stage, but with lower significance. Reviewing literature to explain these sequences in OCT: we found OCT shows more RNFL swelling during acute retrobulbar ON than can be observed in routine fundoscopy with a swelling up to 82 %; obviously due to axoplasmic flow stasis [[13], [14], [15]]. At the clinical onset: pRNFL edema is present, and almost absent in the macula; while, it disappears after 1 month. This is followed by gradual neurodegeneration of mGCC and pRNFL in the next 4 months [16,17].

We found in both eyes (the acute ON and the fellow): RNFLT were about 95 μm. The noted decrease of RNFLT belongs to the fellow eye in our study, can be explained on basis of previous attack in that side or retrograde neurodegeneration.

Our results agree with literatures’ cut-off values. Fisher et al. compared RNFL values between MS patients and healthy subjects, and found that: the mean RNFLT was reduced in MS patients (92 μm) relative to controls (105 μm) [18]. Henderson et al. evaluated 23 patients with acute unilateral ON with serial OCT testing. The RNFLT was significantly increased in ON eyes relative to non-ON eyes at baseline, but then significantly decreased at all later time [19].

We found insignificant temporal RNFL thinning in acute ON eyes in comparison to the fellow eyes. Instead, we found significant correlation of temporal RNFLT to number of retrolaminar demyelinating foci. Our result agrees with literature; as, many authors had found that the distribution of RNFL loss tends to involve the temporal quadrant in MS-ON, whereas it is more diffusely distributed in neuromyelitis optica [20,21]. Puthenparampil et al. found an inverse correlation between the thickness of the temporal RNFL and the ipsilateral optic radiation white matter lesion load in patients with MS-ON [22]. Recent study demonstrated conflicting data about the main focus of RNFL thinning affecting both temporal and nasal quadrants [23]. The preferential affection of temporal RNFL in MS can be explained by presence of small size neurons in papillomacular bundle that easily fail to remyelinate [14].

Imaging of LC and retrolaminar part of optic nerve by conventional OCT is of variable quality due to many artefacts as light attenuation in deeper structures and blood vessels shadowing. By development of enhanced depth imaging and software like adaptive compensation; visibility of LC and retrolaminar part has been established in multiple OCT platforms [24].

Using EDI-OCT in glaucomatous optic nerve imaging is widely used. However, using it in other types of optic neuropathies is limited. We found 2 studies that used it in measuring lamina cribrosa thickness in acute ON with different results [25].

There is mounting evidence to suggest that the demyelination occurs in MS-ON in anterior retrobulbar (retrolaminar). Therefore, an ability to image this part in acute ON will be of interest. Our hypothesis: the retrolaminar imaging during acute attack can show demyelination areas as hyper-reflective foci or aggregating in longitudinal plaques. This hypothesis was based on myelin physical properties and previous trials to image myelin debris ex vivo. Interesting finding is presence of oval or round hyper-reflective materials in the acute ON eyes; that is not present in the fellow eyes, although history of previous ON attacks. This means our finding is linked to myelin debris. Our biomarker is strengthened by previous works on ex vivo myelin imaging and myelin optical properties.

Reviewing literatures, we found imaging of myelinated axons in is either by contrast or label free methods. Label free methods as dMR in vivo is still low resolution [10,26]. Myelin composition is responsible for its high refractive index and optical birefringence. Utilizing label-free polarization-sensitive OCT (PS-OCT) that can detect both scattering and birefringence, imagining of fibers in the brain tissue with micrometer-level resolution, however, the drawbacks of this method are ex vivo and an inevitable loss of resolution due to laser speckle noise [10].

Demyelination and subsequent neuronal loss occurs in MS, where there is a direct autoimmune attack against the myelin sheath. During myelin damage, the fragmented myelin sheaths aggregate and form large, irregular clusters of myelin debris. Experiment by using spectral confocal reflectance (SCoRe) microscopy, which exploits a unique feature of compact myelin that optically reflects incident laser and minimal tissue preparation. Gonsalvez et al. used SCoRe microscopy to quantify changes to compact myelin and myelin debris in the cuprizone-induced murine model of CNS demyelination. They had published photos about appearance of compact myelin and myelin debris aggregates [[26], [27]]. The previous study had indicated that myelin debris can be identified using SCoRe on the basis of its differential reflection pattern. However this method is still ex vivo or trans craniotomy.

However, current imaging techniques of studying compact myelin integrity in vivo still have clear limitations. We assume that our method is simple, non invasive and cost effective in the diagnosis of acute demyelinating ON.

The limitations of this study are few patient numbers, and lack of comparison to healthy control group. The novel findings may be to some extent affected by non blinded examiner in this research. Also, other types of acute ON are not examined by this method. We recommend continuing larger study in the future based on this preliminary result.

### 4.1 What was known before?

Diagnosis of acute ON in MS patients is mainly clinical plus supplementary test. All suggested supplementary tests have a shortage in imaging of anterior optic nerve demyelination. New evolving imaging techniques of myelin sheaths and its debris are still ex vivo.

### 4.2 What this study adds?

We introduced novel method for myelin debris imaging in retrolaminar optic nerve during acute ON; by using EDI-OCT.

## CRediT authorship contribution statement

**Hend Mohammed Safwat:** Writing – original draft, Methodology, Conceptualization. **Sanaa Ahmed Mohamed:** Writing – review & editing, Supervision, Methodology, Investigation. **Ahmed Hassan Elsheshiny:** Writing – review & editing, Resources, Investigation, Data curation.

## Data availability

All data generated or analyzed during this study are available on request.

## Declaration of competing interest

The authors declare that the research was conducted in the absence of any commercial or financial interest.
